# *OsSPL9* Regulates Grain Number and Grain Yield in Rice

**DOI:** 10.3389/fpls.2021.682018

**Published:** 2021-06-02

**Authors:** Li Hu, Weilan Chen, Wen Yang, Xiaoling Li, Cheng Zhang, Xiaoyu Zhang, Ling Zheng, Xiaobo Zhu, Junjie Yin, Peng Qin, Yuping Wang, Bingtian Ma, Shigui Li, Hua Yuan, Bin Tu

**Affiliations:** ^1^Rice Research Institute, Sichuan Agricultural University, Chengdu, China; ^2^College of Agriculture, Forestry and Health, The Open University of Sichuan, Chengdu, China; ^3^Liaoning Rice Research Institute, Liaoning Academy of Agricultural Sciences, Shenyang, China; ^4^State Key Laboratory of Crop Gene Exploration and Utilization in Southwest China, Sichuan Agricultural University, Chengdu, China

**Keywords:** rice, panicle branches, grain number per panicle, grain yield, *OsSPL9*

## Abstract

Rice grain yield consists of several key components, including tiller number, grain number per panicle (GNP), and grain weight. Among them, GNP is mainly determined by panicle branches and spikelet formation. In this study, we identified a gene affecting GNP and grain yield, *OsSPL9*, which encodes SQUAMOSA-PROMOTER BINDING PROTEIN-LIKE (SPL) family proteins. The mutation of *OsSPL9* significantly reduced secondary branches and GNP. *OsSPL9* was highly expressed in the early developing young panicles, consistent with its function of regulating panicle development. By combining expression analysis and dual-luciferase assays, we further confirmed that OsSPL9 directly activates the expression of *RCN1* (rice TERMINAL FLOWER 1/CENTRORADIALIS homolog) in the early developing young panicle to regulate the panicle branches and GNP. Haplotype analysis showed that Hap3 and Hap4 of *OsSPL9* might be favorable haplotypes contributing to high GNP in rice. These results provide new insights on high grain number breeding in rice.

## Introduction

Rice is a staple food and provides energy for more than half of the population in the world. Grain number per panicle (GNP) is an important factor determining grain yield and is associated with panicle branches and spikelet formation in rice. To date, several genes for GNP have been cloned in rice. *IPA1/OsSPL14* (Ideal Plant Architecture 1) and *DEP1* (DENSE AND ERECT PANICLE 1) are crucial regulators of inflorescence branches (Huang et al., 2009; Jiao et al., 2010; Miura et al., 2010). *Gn1a* (*Grain number 1a*), encoding a cytokinin oxidase, negatively regulates cytokinin accumulation in inflorescence meristems and the number of reproductive organs (Ashikari et al., 2005). *TAWAWA1* encodes a nuclear protein that regulates inflorescence development by promoting inflorescence meristem activity and suppressing the phase change to spikelet meristem identity (Yoshida et al., 2013). Lax panicle genes, *LAX1* and *LAX2*, are involved in the process of axillary meristems formation (Komatsu et al., 2003; Tabuchi et al., 2011). Rice TERMINAL FLOWER 1/CENTRORADIALIS homologs, *RCN1* and *RCN2*, play important roles in controlling the timing of phase transition. Overexpression of these two genes delays transition from the branch shoot to the floral meristem state and causes the formation of higher-order branches (Nakagawa et al., 2002). *FZP* (*FRIZZLE PANICLE*) controls the transition from panicle branches to spikelet formation by negatively regulating *APO2* (*RFL/ABERRANT PANICLE ORGANIZATION 2*) and positively regulating the related OsMADS-box genes (Bai et al., 2016). *FON4* (*Floral organ number 4*) genetically interacts with floral homeotic genes and is responsible for regulating meristem size and determinacy of flora. Loss of function of *FON4* caused multi-floret spikelets in rice (Xu et al., 2017; Ren et al., 2019). *MULTI-FLORET SPIKELET1*(*MFS1*) and *MFS2* are involved in determining the fate of spikelet meristem in rice (Ren et al., 2013; Li et al., 2020). These studies showed that the genetic and molecular mechanisms underlying the panicle branches and spikelet formation are involved in a complex regulatory network. Hence, identification and characterization of diverse mutants related to grain numbers are necessary for further understanding of this process in rice.

SQUAMOSA-PROMOTER BINDING PROTEIN-LIKE (SPL) family proteins are plant-specific transcription factors with a conserved SBP domain consisting of 76–80 amino acid residues (Birkenbihl et al., 2005). There are 19 putative SPL genes in rice (Xie et al., 2006). To date, 14 genes have been identified to be involved in different regulatory pathways. For example, several *SPL* genes, such as *OsSPL2*, *OsSPL4*, *OsSPL7*, *OsSPL13*, *OsSPL14*, *OsSPL16*, *OsSPL17*, and *OsSPL18*, directly regulate yield-related traits, including tiller, panicle branches, grain size, and grain shape (Jiao et al., 2010; Luo et al., 2012; Wang et al., 2012, 2015; Si et al., 2016; Yue et al., 2017; Zhang et al., 2017; Dai et al., 2018; Yuan et al., 2019; Hu et al., 2020). In addition, *OsSPL3* and *OsSPL12* regulate crown root development in rice (Shao et al., 2019). Seed-specific overexpression of *OsSPL12* enhances seed dormancy and inhibits pre-harvest sprouting (Qin et al., 2020). *OsSPL8* is involved in the development of ligule, auricle, and panicle branch angle (Lee et al., 2007). *OsSPL10* negatively controls salt tolerance but positively controls trichome formation in rice (Lan et al., 2019). *OsSPL6* controls panicle cell death by repressing the transcriptional activation of the ER stress sensor, *IRE1* (Wang et al., 2018). Overexpression of *OsSPL9* caused Cu accumulation in the shoot of rice seedlings and in the grain after maturation (Tang et al., 2016); moreover, OsSPL9 directly binds the miR528 promoter to regulate antiviral defense and promote flowering under long-day conditions (Yang et al., 2019; Yao et al., 2019). However, it is unclear whether *OsSPL9* is involved in the regulation of yield-related traits in rice.

In this study, we identified a *less grain number 5* (*lgn5*) mutant, through MutMap analysis and a transgenic experiment, and confirmed that the *lgn5* phenotype was controlled by an SPL family transcription factor, *OsSPL9*. Furthermore, *RCN1*, a positive regulator of panicle branches and GNP (Nakagawa et al., 2002; Wang et al., 2015) was identified as a plausible downstream target of *OsSPL9*. The results will enrich the genetic network regulating grain number and the available genetic resources for breeding improvement of grain number.

## Materials and Methods

### Plant Materials and Growth Conditions

The *lgn5* mutant was identified from an ethyl methanesulfonate (EMS) mutant library of *indica* rice variety, *Shuhui498* (R498). A segregation population derived from the cross between *lgn5* and R498 was used for genetic analysis and gene mapping. All plants including the transgenic lines were grown in an experimental field plot of Sichuan Agricultural University (Chengdu, China) during the normal growing seasons.

### Microscopic Observation

Tissues were collected and immediately fixed in a 2.5% (v/v) glutaraldehyde solution overnight and then dehydrated in an alcohol gradient. Scanning electron microscopy (SEM) observations were processed using a JSM-7500F field emission SEM (JEOL, Japan), as previously described in the study by Li et al. (2014).

### Gene Mapping

MutMap was used for gene mapping (Abe et al., 2012). Briefly, plants with the *lgn5* phenotype were selected from the F_2_ population of the cross between *lgn5* and R498, which were identified as recessive individuals. The DNA of 25 F_2_ plants with *lgn5* was extracted and mixed in an equal proportion, and the mixed DNA was subjected to whole-genome sequencing. The DNA of R498 was re-sequenced as a control. Then, these short reads obtained from mutant-type plants and R498 were aligned to the reference genome sequence (*Nipponbare*).

### CRISPR/Cas9 Vector Construction and Rice Transformation

To knock out *OsSPL9*, we designed the target site at the third exon of *OsSPL9* (Xie et al., 2017), and the target sequence was cloned into a sgRNA expression cassette driven by the *OsU6a* promoter. The sgRNA cassette was recombined into the pYLCRISPR/Cas9Pubi-H vector as previously described by Shan et al. (2013). The final CRISPR/Cas9 construct was introduced into *japonica* variety, Zhonghua11 (ZH11), by *Agrobacterium tumefaciens*-mediated transformation (Jeon et al., 2000). For mutation detection, DNA of T0 transgenic plants was extracted and the target region was amplified by PCR for sequencing. The primers for vector construction and detection are listed in Supplementary Table 1.

### RNA Extraction and Reverse Transcription Quantitative PCR Analysis

Total RNA was extracted from different rice tissues (root, stem, leaf blade, leaf sheath, young panicle, hull, and endosperm) using plant RNA Kit I (OMEGA Bio-Tek, Norcross, United States). cDNA was synthesized from 500 ng of total RNA using a reverse transcription kit (TaKaRa, Dalian, China). RT-qPCR analysis was performed using the SYBR Green Real-Time PCR Mix (KAPA, Boston, United States) on a CFX96™ Real-Time PCR system (Bio-Rad, CA, United States). The *ACTIN* gene (*LOC_Os03g50885*) was used as an internal control. The primer sequences used for RT-qPCR analysis are listed in Supplementary Table 1.

### GUS Staining

A 2.5 Kb region upstream of *OsSPL9* was amplified and cloned into the vector DX2181 to generate the *proOsSPL9::GUS* construct. The construct was transformed into ZH11 by *A. tumefaciens*-mediated transformation (Jeon et al., 2000). Various rice tissues were collected and submerged into the GUS staining solution [50 mM Na3PO4, pH 7.2, 1 mM K4Fe(CN)6, 1 mM K3Fe(CN)6, 1 mM 5-bromo-4-chloro-3-indolyl-β-d-glucuronic acid, and 0.1% (v/v) Triton X-100] and then incubated at 37°C for 12–24 h. The stained tissues were transferred to 70% (v/v) ethanol for decolorization. Photographs were taken using a a MICROTEK ScanMaker i800 plus scanner (Shanghai, China).

### Dual-Luciferase Assay

The transcriptional activation of *RCN1* by OsSPL9 was performed using the dual-luciferase assay. The full-length CDS of OsSPL9 was amplified and inserted into the pGreen II 62-SK driven by a CaMV *35S* promoter for the effector construct. A fragment size of 2,000 bp of *RCN1* promoter was inserted into the pGreen II 0800-LUC (LUC) vector to generate the reporter construct. Renilla luciferase (REN) driven by the CaMV *35S* promoter in the same construct was used as an internal control. The effector and reporter constructs were transiently co-expressed in rice protoplasts. LUC and REN activities were measured using a Dual-Luciferase Reporter Assay System (Promega, United States) according to the instructions of the manufacturer. The primer sequences used for vector construction are listed in Supplementary Table 1.

### Haplotype Analysis

The single nucleotide polymorphism (SNP) and insertion/deletion (InDel) information of the *OsSPL9* genome in rice germplasms and the grain number of Hap1–Hap4 haplotypes were obtained from RiceVarMap v2.0.^1^ Haplotype analysis was performed based on the non-synonymous variations.

## Results

### Characterization of the *lgn5* Mutant

**Figure 1:**
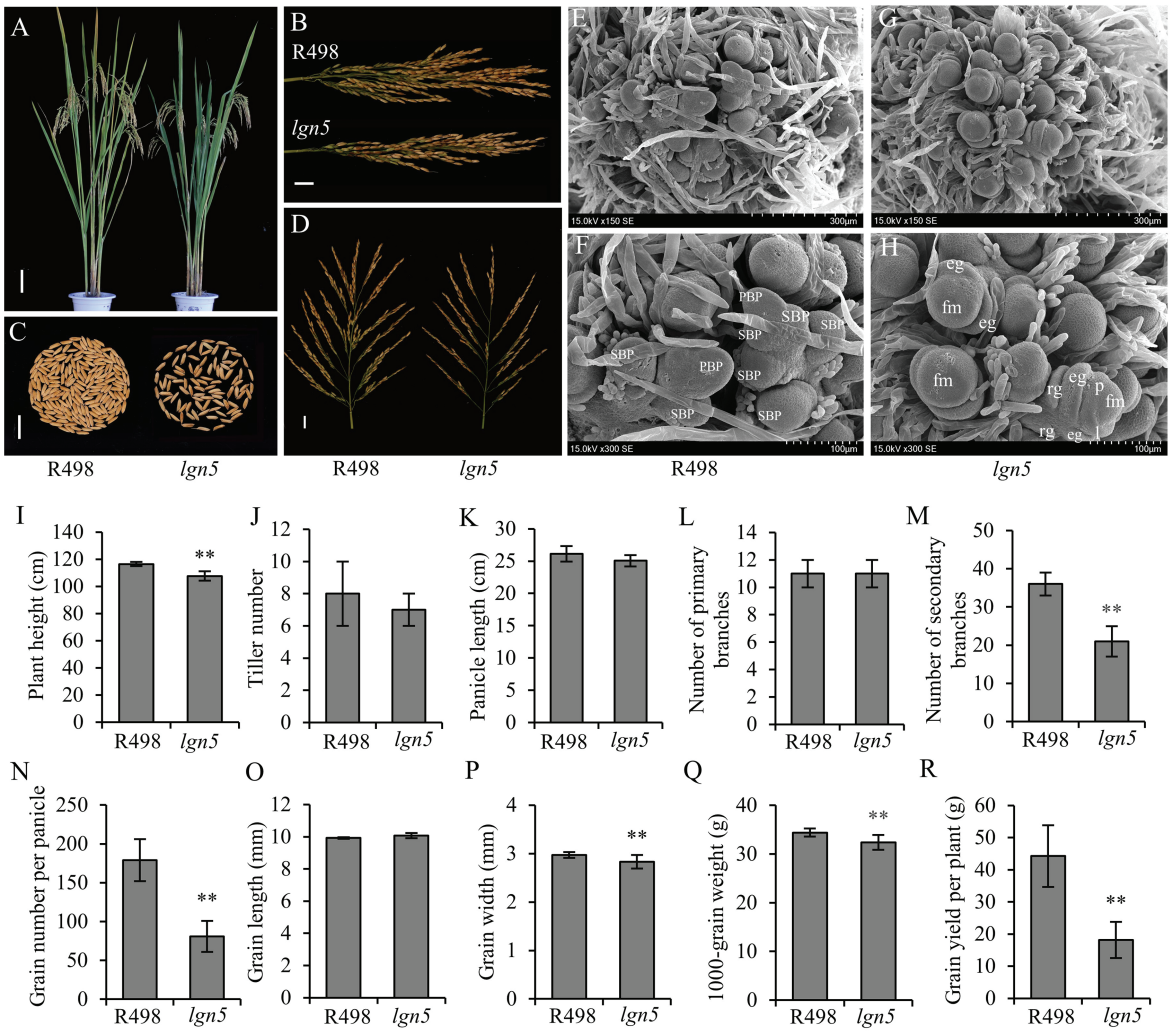
Phenotype of *less grain number 5* (*lgn5*) mutant. **(A)** Plant morphology of R498 and *lgn5* during grain filling. Scale bar: 10 cm. **(B)** Panicles of R498 and *lgn5*. Scale bar: 2 cm. **(C)** Comparison of grain number per panicle (GNP) between R498 and *lgn5*. Scale bar: 2 cm. **(D)** Panicle morphology of R498 and *lgn5*. Scale bar: 2 cm. **(E–H)** Scanning electron microscopy (SEM) observation of the young panicle in the early developmental stage between R498 and *lgn5*. **(E,G)** Young panicle of 0.5–1 mm. **(F,H)** Enlarged views of **(E,G)**, respectively. PBP, primary branch primordia; SBP, secondary branch primordia; fm, floral meristem; p, palea; l, lemma; eg, empty glume; rg, rudimentary glume. Scale bar: 300 μm **(E,G)** and 100 μm **(F,H)**. **(I–R)** Statistical analysis of major agronomic traits in R498 and *lgn5*. Data are given as means ± SD of five biological replicates. ** indicates *p* < 0.01 by Student’s *t*-test.

The *lgn5* mutant was isolated from an EMS mutant library of *indica* rice variety, *Shuhui498* (R498), which is an excellent restorer of hybrid rice. Compared with R498, the plant height of *lgn5* was reduced by 7.6% (Figures 1A,I), but there was no significant difference in the tiller number and panicle length of *lgn5* (Figures 1B,J,K). Similarly, there was no significant difference in the number of primary branches; however, the number of secondary branches was significantly reduced by 41.7%, leading to a 54.7% reduction in GNP (Figures 1C,D,L–N). The grain length had no significant change, but the grain width decreased by 4.7%, leading to a 5.8% reduction in 1,000-grain weight (Figures 1O–Q). Finally, the grain yield per plant was reduced by 59% (Figure 1R).

To understand the cytological mechanism of panicle development in *lgn5*, we performed SEM observation. When the length of young panicles was 0.1–0.5 mm, R498 plants had a large number of secondary branch primordia, while *lgn5* plants were already in the stage of spikelet primordia differentiation (Figures 1E–H). This result suggested that the panicle development of *lgn5* was earlier than that of R498. Taken together, these results indicated that the decrease of secondary branches in *lgn5* might be caused by the shortened differentiation period of secondary branch primordia and the early formation of spikelets.

### *OsSPL9* Is Responsible for the *lgn5* Phenotype

To identify the gene responsible for the *lgn5* phenotype, *lgn5* was backcrossed with the parental line R498. Approximately, one-quarter of the F_2_ plants (χ^2^_c_ = 0.260 < χ^2^_0.05,1_ = 3.84) had fewer grains (Supplementary Figure S1A), indicating that a single recessive allele was responsible for the *lgn5* phenotype. Then, we performed gene mapping using the MutMap strategy and identified a linkage region on chromosome 5 (Supplementary Figures S1B,C). Three linked SNPs with the SNP index of 1 on chromosome 5 were identified in *LOC_Os05g31480*, *LOC_Os05g31830*, and *LOC_Os05g33810*. Among these SNPs, SNP1 in *LOC_Os05g31480* was located in an intron; SNP2 in *LOC_Os05g31830* led to a synonymous mutation; and SNP3 in *LOC_Os05g33810* caused a non-synonymous mutation (Glu561Lys) in an exon (Supplementary Figure S1D). Therefore, *LOC_Os05g33810*, which encodes the SPL family transcription factor, OsSPL9, was the likely candidate gene for the *lgn5* phenotype. Phylogenetic analysis of OsSPL9 in different plant species revealed that OsSPL9 was conserved in *Poacea* (Supplementary Figure S2). In addition, amino acid substitution in the *lgn5* mutant was highly conserved among different plant species (Supplementary Figure S3), indicating that this amino acid may be an essential component for OsSPL9 function.

**Figure 2:**
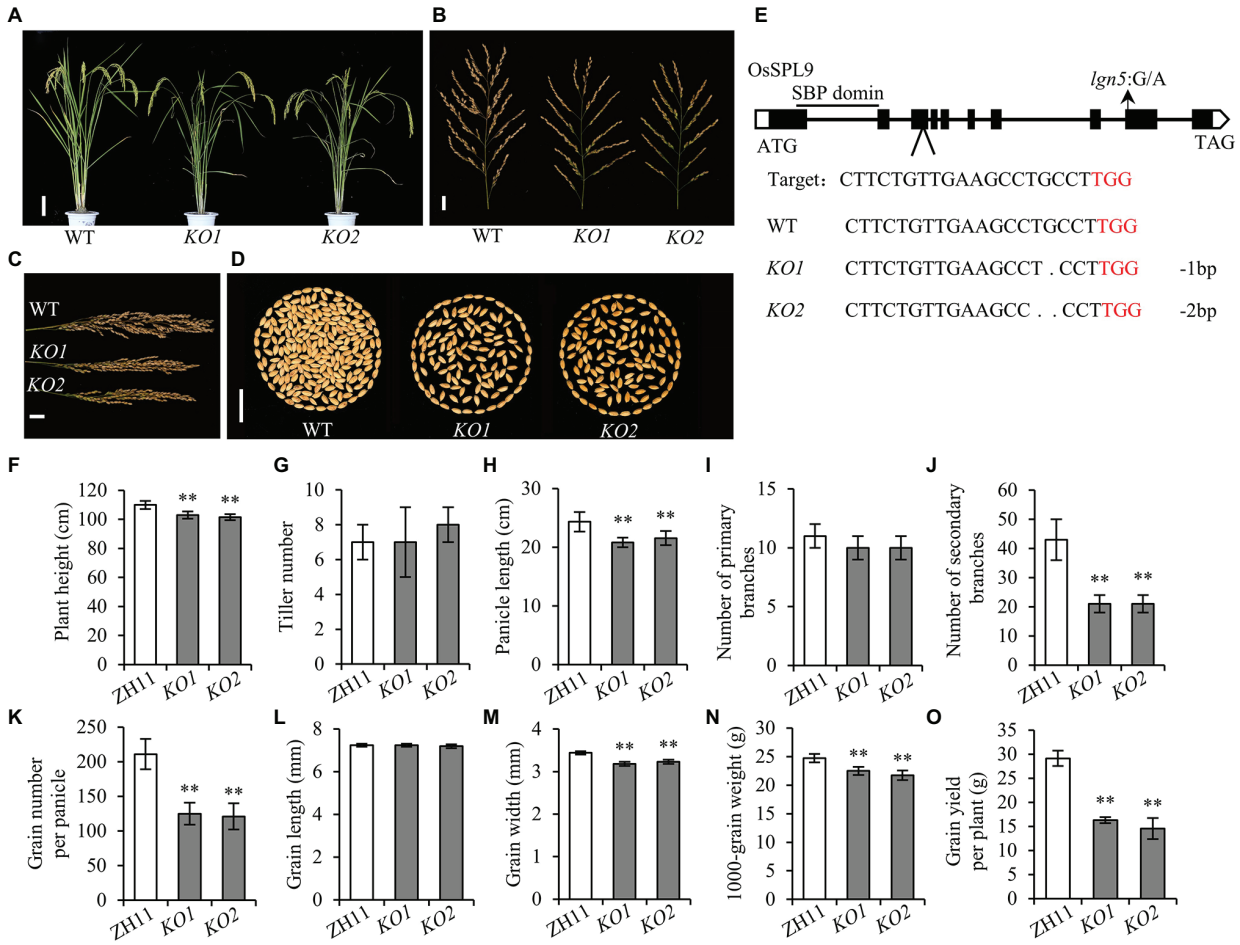
Phenotypic comparison of wild type and CRISPR mutants. **(A)** Plant morphology of wild type and CRISPR mutants. Scale bar: 10 cm. **(B)** Panicle morphology of wild type and CRISPR mutants. Scale bar: 2 cm. **(C)** Panicles of wild type and CRISPR mutants. Scale bar: 2 cm. **(D)** Comparison of GNP between wild type and CRISPR mutants. Scale bar: 2 cm. **(E)** Schematic diagram of the target site in *OsSPL9* and the target sequence alignment of wild type and CRISPR mutants. The number of altered bases is indicated on the right; “−” indicates deleted bases. ATG, start codon; TAG, stop codon; the arrow shows the mutant site in *lgn5*. **(F–O)** Agronomic traits in wild type and CRISPR mutants. Data are given as means ± SD of five biological replicates. ** indicates *p* < 0.01 by the Student’s *t*-test.

To further confirm that the *lgn5* phenotype was caused by the mutation of *OsSPL9*, we obtained knockout (*KO*) mutant plants of *OsSPL9* in the Zhonghua11 (ZH11) background using the CRISPR/Cas9 genome editing system (Shan et al., 2013). Two independent homozygous *KO* mutants with different mutation types were generated, and both mutations caused premature stop codons (Supplementary Figure S4). Compared with wild-type ZH11 plants, the plant height and the panicle length of *KO* mutants were significantly decreased (Figures 2A,C,F,H). The number of primary branches did not differ, but the number of secondary branches reduced by 51.2% on average, and the GNP decreased by 41.7% (Figures 2B,D,I–K). The grain length was not significantly different, but the grain width and 1,000-grain weight were reduced by 6.8 and 10.6% on average, respectively (Figures 2L–N). The reduction in both grain number and grain weight caused a 47% reduction in grain yield per plant in knockout mutants of *OsSPL9* (Figure 2O). Taken together, knockout mutants of *OsSPL9* showed similar phenotype variations as the *lgn5* mutant, indicating that the SNP3 in *OsSPL9* is responsible for the *lgn5* mutant phenotype.

### Expression Pattern of *OsSPL9*

**Figure 3:**
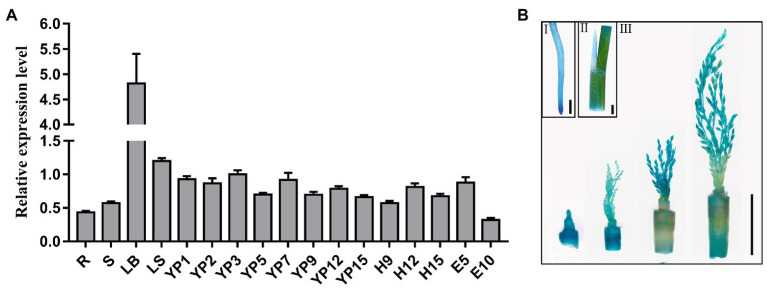
Expression pattern of *OsSPL9*. **(A)** Transcript levels of *OsSPL9* in various organs by RT-qPCR. R, young root; S, stem; LB, leaf blade in the tillering stage; LS, leaf sheath; YP1–YP15, young panicles of different lengths (1–15 cm). H9–H15, hulls from panicles with different lengths (9–15 cm). E5–E10, endosperm at 5 and 10 days after fertilization. Data are given as means ± SD of three biological replicates. **(B)** GUS staining in the *ProOsSPL9*::GUS transgenic plants. Root (I); leaf sheath (II); and early developing young panicle and stem (III). Scale bars: 2 mm (I,II) and 1 cm (III).

We analyzed the spatial expression of *OsSPL9* by RT-qPCR. *OsSPL9* was expressed in all of the investigated organs and was highly expressed in leaf blades and developing young panicles (Figure 3A). GUS staining was further performed in the *ProOsSPL9*::GUS transgenic plants. GUS activity was detected predominantly in the root, leaf sheath, stem, and developing young panicles (Figure 3B). These results indicate that *OsSPL9* is constitutively expressed, and the high expression level in young panicles supported its role in the regulation of panicle development and grain number.

### *OsSPL9* Positively Regulates the Expression of *RCN1*

**Figure 4:**
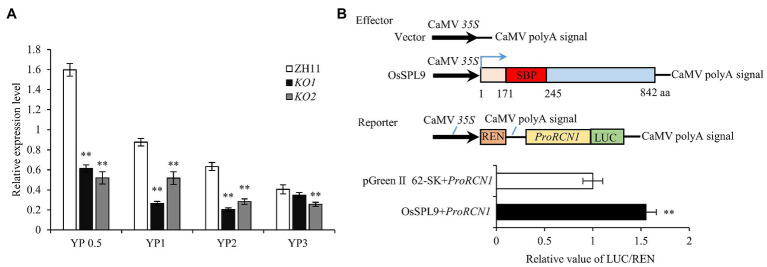
OsSPL9 activates the transcription of rice TERMINAL FLOWER 1/CENTRORADIALIS homolog (*RCN1*). **(A)** The transcriptional levels of *RCN1* were downregulated in young panicles in *KO* mutants. YP0.5, YP1, YP2, and YP3, young panicles of 0.5, 1, 2, and 3 cm in length, respectively. Data are given as means ± SD of three biological replicates. ** indicates *p* < 0.01 by Student’s *t*-test. **(B)** Luciferase transient transcriptional activity assay in rice protoplasts to show that OsSPL9 activates *RCN1* promoter. The pGreen II 0800-LUC/Renilla luciferase (LUC/REN) ratio of empty vector and *proRCN1*::LUC was normalized to 1. Data are given as means ± SD of three biological replicates. ** indicates *p* < 0.01 by Student’s *t*-test.

As the mutants of *OsSPL9* (*lgn5* and *KO* lines) showed a significant decrease in grain number, we detected the expression level of the genes related to grain number in the early developing young panicles of the *KO1* line. The results showed that the expression level of most tested genes (including *DST*, *FZP*, *OsGRF6*, *GNP1*, *Gn1a*, *DEP1*, *DEP2*, *DEP3*, *OsCLV1*, *LAX1*, and *LAX2*) had no significant difference between wild type and *KO1*; however, *RCN1*, a positive regulator of panicle branches in rice (Nakagawa et al., 2002; Wang et al., 2015), was significantly downregulated in both *KO* and *lgn5* mutants (Figure 4A; Supplementary Figure S5). Overexpression of *RCN1* promotes the production of higher-order branches and spikelets (Nakagawa et al., 2002; Wang et al., 2015); hence, *OsSPL9* might regulate panicle branches and grain number through *RCN1*. Consistent with this hypothesis, sequence analysis showed five GATC motifs in the promoter region (from −2000 to −1 bp) of *RCN1* (Supplementary Figure S6), which was the recognition sequence of SPL protein for DNA binding (Birkenbihl et al., 2005). Therefore, we performed dual-luciferase assays in rice protoplasts to test whether OsSPL9 could directly bind to the promoter of *RCN1*. The results showed that OsSPL9 significantly enhanced the expression of the LUC reporter gene (Figure 4B). Thus, the results indicated that *OsSPL9* regulates panicle branches and grain numbers by upregulating *RCN1* expression in rice.

### Haplotype Analysis of *OsSPL9*

**Figure 5:**
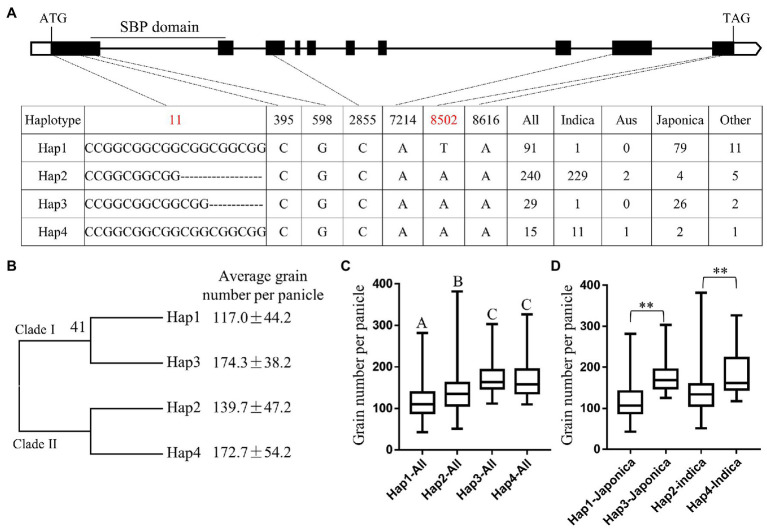
Haplotype analysis of *OsSPL9* in rice accessions. **(A)** Single nucleotide polymorphism (SNP) divergence in the coding region of *OsSPL9*. **(B)** Phylogenetic analysis of *OsSPL9* coding region using the neighbor-joining method. **(C)** The average GNP of *OsSPL9* haplotypes in cultivars. Data are means ± SD. *n* = 91, 240, 29, and 15. The letters above the error bars indicate significant differences at *p* < 0.01 using multiple comparisons. **(D)** Comparison of the average GNP of *OsSPL9* haplotypes in the same subspecies. Data indicate means ± SD. *n* = 79, 26, 229, and 11. Significant differences are determined using Student’s *t*-test. ** indicates *p* < 0.01.

We analyzed the genome variation of *OsSPL9* in 4,726 rice accessions using Rice Variation Map v2.0, (see footnote 1) and 12 polymorphisms were detected in the exons of *OsSPL9*; however, only seven polymorphisms were non-synonymous (Supplementary Table 2). We used these seven non-synonymous polymorphisms for haplotype analysis in 375 cultivars with different GNP and found that five of the polymorphisms (sites in 395, 598, 2,855, 7,214, and 8,616) were identical in these cultivars, and four haplotypes (Hap1–Hap4) were identified based on two polymorphisms (sites in 11 and 8,502; Figure 5A). Phylogenetic analysis showed that the four haplotypes were divided into two clades: the clade I containing Hap1 and Hap3 was mainly found in *japonica* cultivars, and the clade II containing Hap2 and Hap4 was mainly present in *indica* cultivars (Figures 5A,B), indicating the divergence of *OsSPL9* between *japonica* and *indica* cultivars. Furthermore, we compared the GNP between these haplotypes and found that Hap3 and Hap4 had the highest GNP, and Hap1 had the lowest GNP (Figures 5B,C). To exclude the influence of the genetic background of each haplotype, we also compared the GNP of different haplotypes in the same subspecies. For example, in the same *japonica* background, Hap3 had 50.5% higher GNP than Hap1 on average; and in the same *indica* background, Hap4 had 34.4% higher GNP than Hap2 on average (Figure 5D). Taken together, these results indicated that Hap3 and Hap4 of *OsSPL9* might be favorable haplotypes.

## Discussion

### *OsSPL9* Plays an Important Role in the Regulation of Grain Number

There are 19 putative SPL genes in rice (Xie et al., 2006), and 14 genes have been identified to be involved in various biological processes during rice growth and development, such as regulating tiller, panicle branches, grain size, the development of crown root, ligule, auricle and trichome, and stress response (Lee et al., 2007; Jiao et al., 2010; Luo et al., 2012; Wang et al., 2012, 2015; Si et al., 2016; Yue et al., 2017; Zhang et al., 2017; Dai et al., 2018; Lan et al., 2019; Shao et al., 2019; Yuan et al., 2019; Hu et al., 2020). *OsSPL9* was proven to regulate Cu uptake and distribution by directly activating the expression of Cu transporter genes in rice (Tang et al., 2016), and an *OsSPL9*-miR528 pathway was involved in the regulation of plant resistance to rice *stripe virus* and rice flowering under long-day conditions (Yang et al., 2019; Yao et al., 2019). However, there has not been a comprehensive investigation on the function of the *OsSPL9* on yield-related traits. In this study, we investigated the genetic effects of *OsSPL9* on agronomic traits in the *indica* and *japonica* backgrounds and found that the number of secondary branches and GNP were significantly reduced in the mutants of *OsSPL9* in both *indica* (R498) and *japonica* (ZH11) backgrounds (Figures 1, 2), indicating that *OsSPL9* plays an important role in the regulation of grain number. Consistent with its function, *OsSPL9* was expressed in the early developing young panicles (Figure 3B).

Moreover, haplotype analysis showed that Hap3 and Hap4 of *OsSPL9* might be favorable haplotypes, and considering that both Hap3 and Hap4 accounted only for a relatively lower proportion (7.7 and 4.0%, respectively) of these cultivars (Figure 5), they might have a great potential for higher grain number breeding in rice. In future, it will be of great significance to reveal the functional differences of different haplotypes at the biochemical, molecular, and genetic levels. Especially, the polymorphisms located at 11 and 8,502, which, respectively, led to amino acid in-frame deletion and substitution (Supplementary Table 2), might be responsible for biological functional differences.

### The C-Terminal Region of OsSPL9 Is Essential for Its Biological Function

SQUAMOSA-PROMOTER BINDING PROTEIN-LIKE family proteins are plant-specific transcription factors. They all contain the highly conserved SBP-domain, which is necessary for DNA binding and nuclear localization (Birkenbihl et al., 2005). In addition, the N-terminus or C-terminus of SPL proteins exhibits transcriptional activation activity. For example, the activation domain of OsSPL16/OsSPL9 is located at the N-terminus (Wang et al., 2012; Tang et al., 2016), while the activation domain of OsSPL14 is located in the C-terminus region (Lu et al., 2013). Both N-terminus and C-terminus of OsSPL18 have transcriptional activation activity (Yuan et al., 2019). In this study, although the amino acid substitution in *lgn5* mutant and the premature protein of *KO* mutants of OsSPL9 were located at the C-terminus without disrupting its N-terminal activation domain and SBP-domain (Figure 2E; Supplementary Figure S4), both *lgn5* and *KO* mutants of *OsSPL9* exhibited significantly decreased GNP and grain yield (Figures 1, 2). Thus, the results genetically demonstrated that the C-terminal region of OsSPL9 is essential for its biological function.

### The OsSPL9-*RCN1* Pathway Regulates Grain Number in Rice

Rice TERMINAL FLOWER 1/CENTRORADIALIS homolog was an important positive regulator of panicle branches (Nakagawa et al., 2002; Wang et al., 2015), and its expression level was significantly downregulated in the early stage of developing young panicles in the mutants of *OsSPL9* (Figure 4A; Supplementary Figure S5), which was consistent with the decreased panicle branches and grain number in *lgn5* and *KO* mutants (Figures 1, 2). Further, dual-luciferase assays confirmed that OsSPL9 could directly activate *RCN1* expression (Figure 4B). In addition, the cytological analysis showed that *OsSPL9* regulated panicle development by affecting the phase transition from branch primordia to the spikelet primordia state (Figures 1E–H), which is similar to that of *RCN1* (Nakagawa et al., 2002). Therefore, the results revealed a plausible OsSPL9-*RCN1* pathway regulating panicle branches and grain number in rice.

## Data Availability Statement

The original contributions presented in the study are included in the article/Supplementary Material, further inquiries can be directed to the corresponding authors.

## Author Contributions

LH and WC conceived the research, performed most of the experiments, and wrote the manuscript. WY, XL, and CZ were responsible for the identification and characterization of mutants. XZha, LZ, XZhu, and JY completed part of the work. PQ, YW, and BM provided the instructions about the experiments. SL, HY, and BT helped to review and revise the manuscript. All authors contributed to the article and approved the submitted version.

### Conflict of Interest

The authors declare that the research was conducted in the absence of any commercial or financial relationships that could be construed as a potential conflict of interest.

## Footnotes

^1^http://ricevarmap.ncpgr.cn/v2/
