# Supplementary material for: OsSPL9 Regulates Grain Number and Grain Yield in Rice
Source: Front Plant Sci. 2021 Jun 2;12:682018. doi: 10.3389/fpls.2021.682018 (PMC8207197; doi:10.3389/fpls.2021.682018)
Supplement: Supplementary file 1 [file Table_1.DOCX]

**Supplementary Table 1. Primers used in this study.**

| Purpose | Primer name | Primer sequence (5'-3') |
| --- | --- | --- |
| Genotype identification of *lgn5* mutant (4-pair-primer-pcr) | Forward inner primer (A allele) | TTCTATGATTTTATTTTGCAGGGGA |
|  | Reverse inner primer (G allele) | ATTTGATAGCCCAAACATGTTGTC |
|  | Forward outer primer | AATGCACATAAGCAATCATTTGC |
|  | Reverse outer primer | ATTAGCCATCCAATGTCGATGA |
| pYLCRISPR/Cas9 Pubi-H construct for *OsSPL9* | *OsU6a*-target-F | GCCGCTTCTGTTGAAGCCTGCCT |
|  | *OsU6a*-target-R | AAAC AGGCAGGCTTCAACAGAAG |
| Detection the knock-out mutants of *OsSPL9* | *OsSPL9*-F | GCCTGTTTACTTCTGTTGTC |
|  | *OsSPL9*-R | CCTGGTTATATACTTGGTGG |
| GUS constructs | *ProOsSPL9::GUS*-F | TGATCTACAGCGCTGAAGCTTAGTTGGTGAACAAAATTTCAAAGCT |
|  | *ProOsSPL9::GUS*-R | GGACTGACCACCCGGGGATCCCCCCGGTGGGCCACCCCC |
| Dual-luciferase assay | *ProRCN1::LUC*-F | cttgatatcgaattcctgcagTAAATCCTATCCTAGTCAGCACACATG |
|  | *ProRCN1::LUC*-R | cgctctagaactagtggatccGTTTAGAGGAGCAGACAGTAATATTTTGC |
|  | *35S:OsSPL9*-F | cgctctagaactagtggatccATGGACGCCCCCGGCGGC |
|  | *35S:OsSPL9*-F | cttgatatcgaattcctgcagCTATGATGAGTAGTTCCTAGACAAGTACCTC |
| Reverse transcription quantitative PCR (RT-qPCR) | *OsSPL9-*RT-F | AGATGGGCAGGTGATTAT |
|  | *OsSPL9-*RT-R | TGTGGGAGAGCTTTAGTC |
|  | *DST-*RT-F | ATGATCGACATGCTCAACTG |
|  | *DST-*RT-R | GTTCTCCTCGCCGTTGTT |
|  | *FZP-*RT-F | GCATGGCTAATCACGCACTTT |
|  | *FEP-*RT-R | CCAAGCCACTCTTCTTGTTCG |
|  | *OsGRF6-*RT-F | CATCAGCCTCAACATCTCC |
|  | *OsGRF6-*RT-R | TCCAGTTTGCTTCTCCCT |
|  | *GNP1-*RT-F | TACGCCAGCAGCTTCACGG |
|  | *GNP1-*RT-R | TCCATCAGCTCCAGCGACA |
|  | *Gn1a-*RT-F | GATAGCCTACAAGCAGTA |
|  | *Gn1a-*RT-R | GCCTTTGGATCATACTTG |
|  | *DEP1-*RT-F | CCGTTTCTCGTTCTGGAT |
|  | *DEP1-*RT-R | ATCTGTGCCTCCTTCTCT |
|  | *DEP2-*RT-F | TGCGTGATAGCCTAGAACGAAG |
|  | *DEP2-*RT-R | CTGGAATCAGCACTCCTGGATG |
|  | *DEP3-*RT-F | TGGTGGACCAAGCTGTATCA |
|  | *DEP3-*RT-R | ATCACCTTCTTCCCCCTGAA |
|  | *OsCLV1-*RT-F | ACATGCTCTCCAACCCAAACTCG |
|  | *OsCLV1-*RT-R | TAGCCGCTAATTAAGCGCACAC |
|  | *OsCLV2-*RT-F | CGAACAACCGAATATCTG |
|  | *OsCLV2-*RT-R | GAACGAGAACATCCAATC |
|  | *RCN1-*RT-F | GACCTGCGATCTTTCTTCAC |
|  | *RCN1-*RT-R | GACAATTGGAGCTGCATTTC |
|  | *LAX1-*RT-F | GATGACGACGCTGGAGAT |
|  | *LAX1-*RT-R | GACATTGCACACCGAGTAG |
|  | *LAX2-*RT-F | GCCATCCACTACGTCAAGT |
|  | *LAX2-*RT-R | TGGACGAAGACACAGCAAGG |
