# Supplementary material for: OsSPL9 Regulates Grain Number and Grain Yield in Rice
Source: Front Plant Sci. 2021 Jun 2;12:682018. doi: 10.3389/fpls.2021.682018 (PMC8207197; doi:10.3389/fpls.2021.682018)
Supplement: Supplementary file 3 [file Data_Sheet_1.docx]

**Supplementary Material**


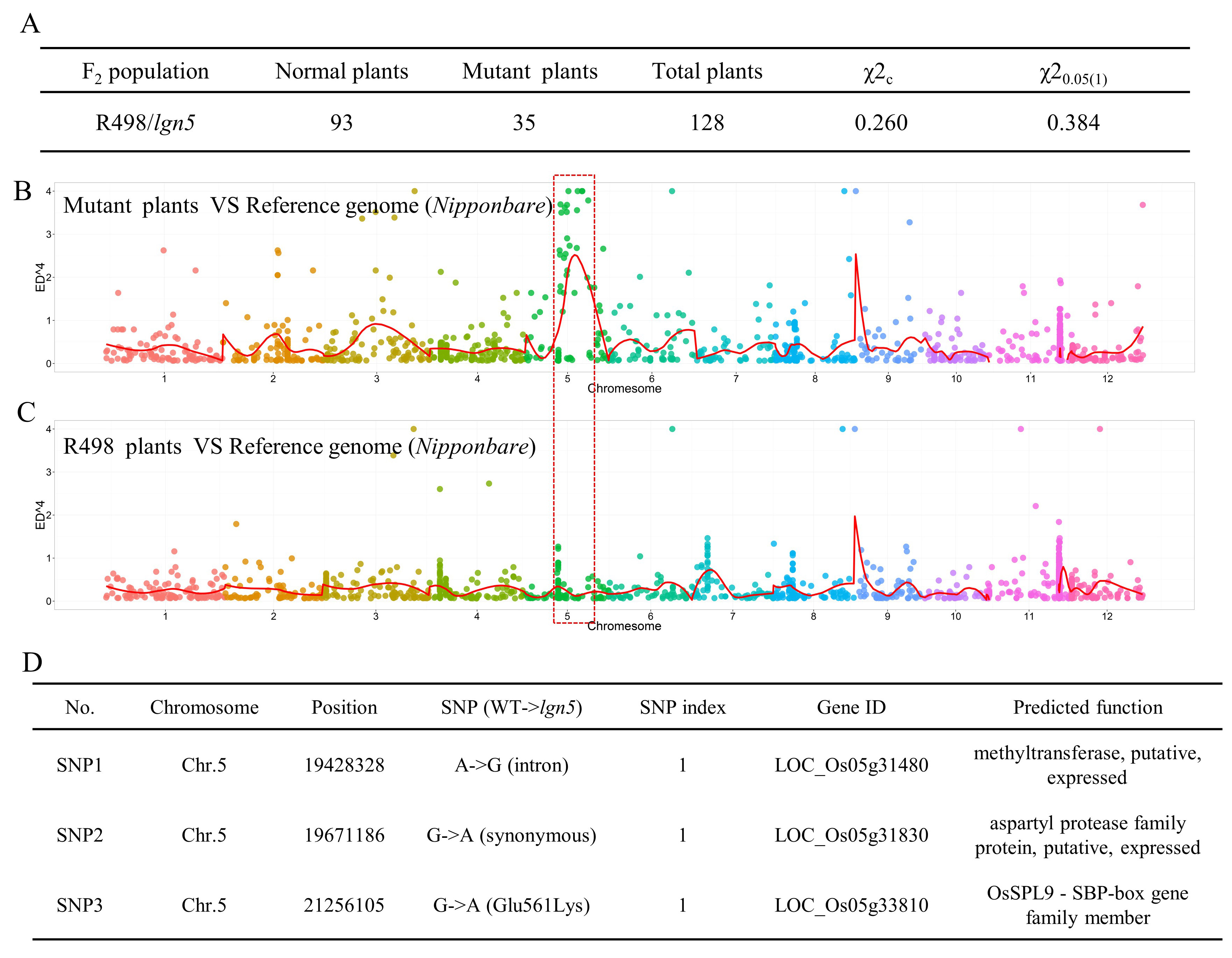


**Supplementary Figure S1. Identification of the SNPs causing the *lgn5* phenotype using MutMap method.**

**(A)** Genetic analysis of lgn5. **(B-C)** Euclidean distance (ED) scores across the genome with the fourth power. The red dotted box shows the linkage region on chromosome 5. **(D)** Three SNPs on chromosome 5 with SNP index of 1.


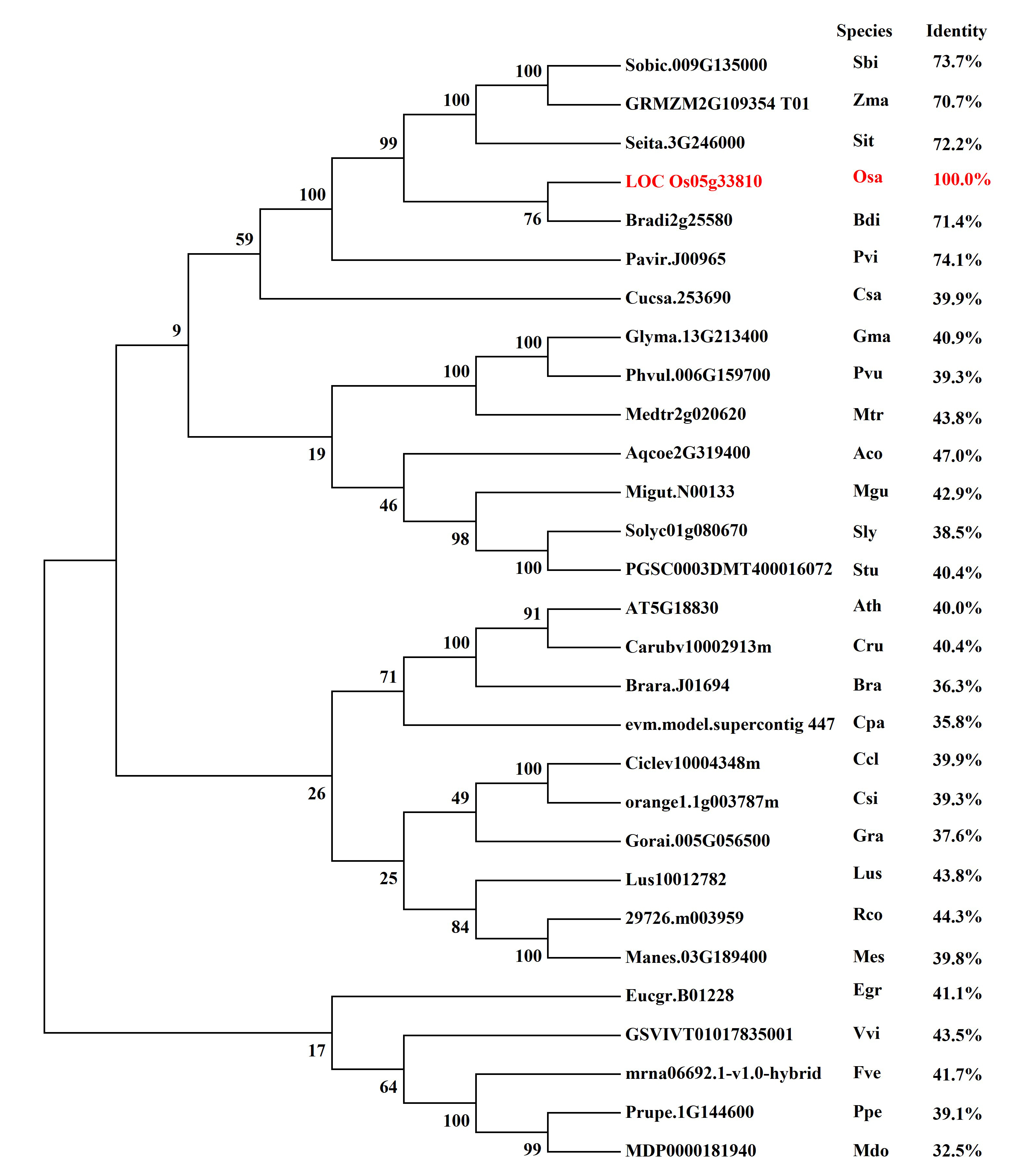


**Supplementary Figure S2. A neighbor-joining tree of OsSPL9 and its homologs in plant.**

All the protein sequences were obtained from phytozome v9.1 (http://www. phytozome.net). A neighbor-joining tree was generated using MEGA5.1. Numbers on the tree indicate bootstrap support based on 1000 replicates. Sbi, *Sorghum bicolor*; Zma, *Zea mays*; Sit, *Setaria italic*; Osa, *Oryza sativa*; Bdi, *Brachypodium distachyon*; Pvi, *Panicum virgatum*; Csa, *Cucumis sativus*; Gma, *Glycine max*; Pvu, *Phaseolus vulgaris*; Mtr, *Medicago truncatula*; Aco, *Aquilegia coerulea*; Mgu, *Mimulus guttatus*; Sly, *Solanum lycopersicum*; Stu, *Solanum tuberosum*; Ath, *Arabidopsis thaliana*; Cru, *Capsella rubella*; Bra, *Brassica rapa*; Cpa, *Carica papaya*; Ccl, *Citrus clementina*; Csi, *Citrus sinensis*; Gra, *Eucalyptus grandis*; Lus, *Linum usitatissimum*; Rco, *Ricinus communis*; Mes, *Manihot esculenta*; Egr, *Eucalyptus grandis*; Vvi, *Vitis vinifera*; Fve, *Fragaria vesca*; Ppe, *Prunus persica*; Mdo*, Malus domestica*.


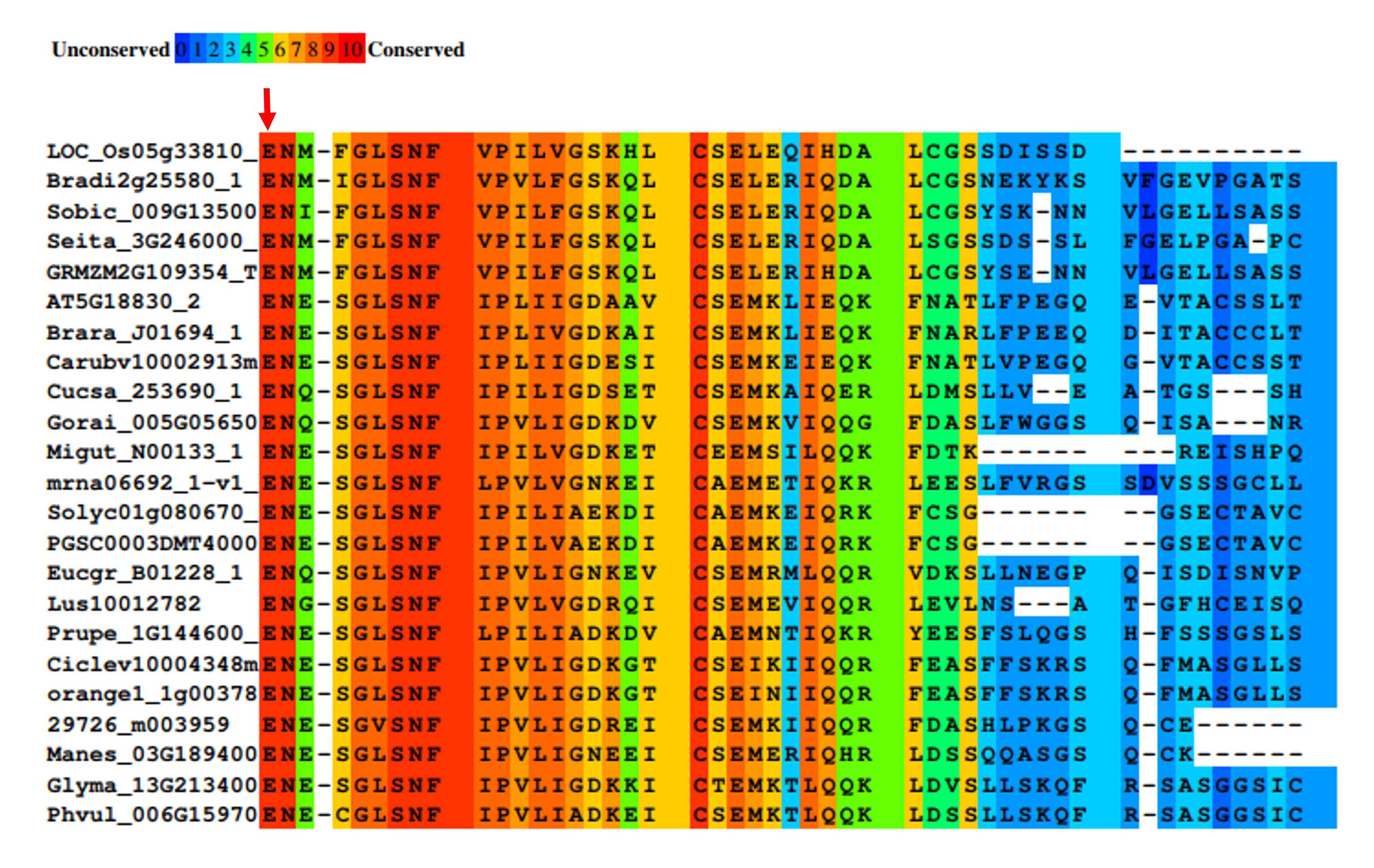


**Supplementary Figure S3. Mutation site in *lgn5* was conserved in different plants.**

The red arrow indicates the substitution of amino acid in *lgn5* mutant. All the amino acid sequence obtained from http://www.phytozome.net.


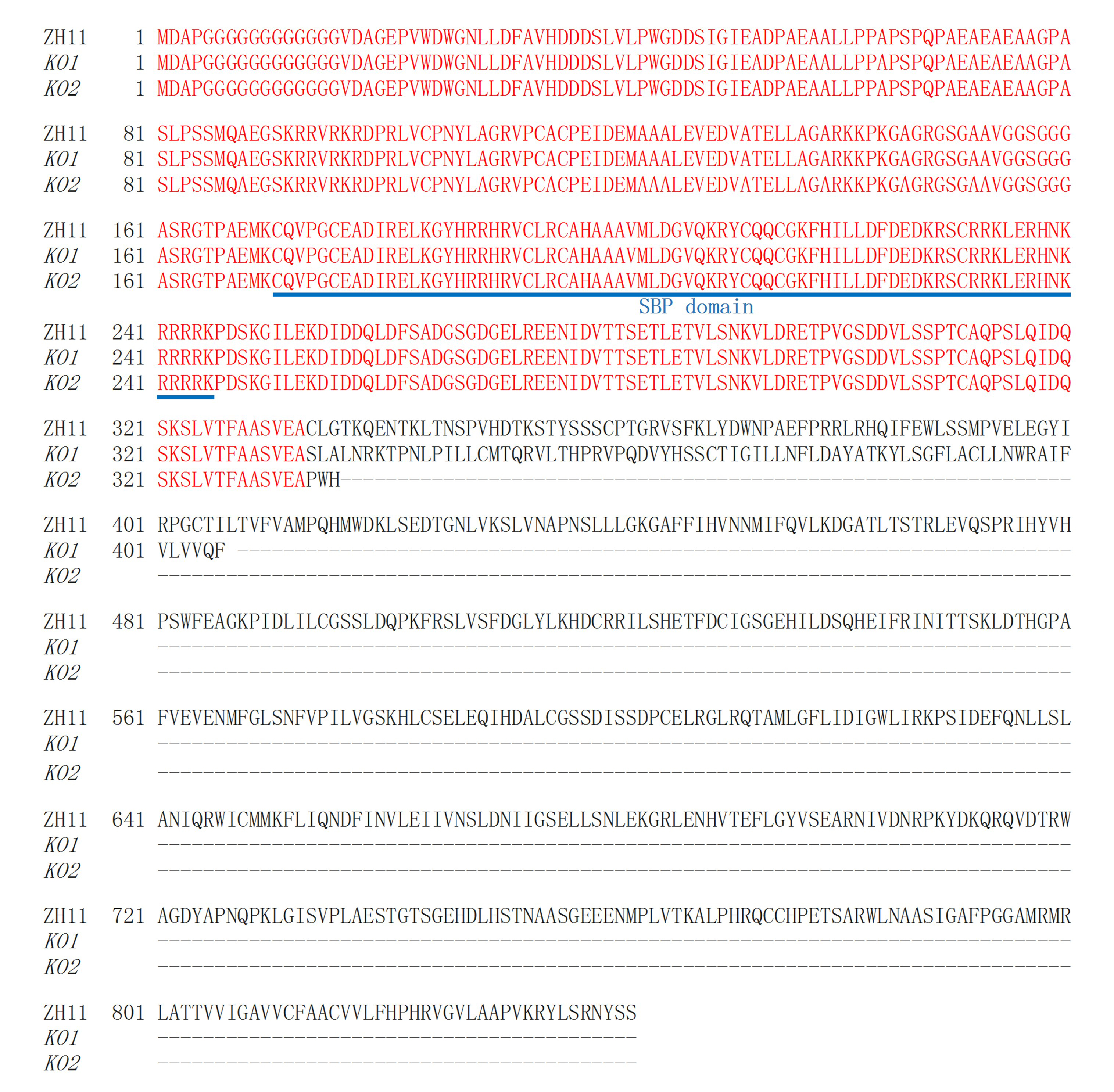


**Supplementary Figure S4. Amino acid sequence alignment of OsSPL9 between ZH11 and knockout (*KO*) lines.**

The numbers on the left show the position of amino acid residues in the protein. The SBP domain of OsSPL9 was showed.


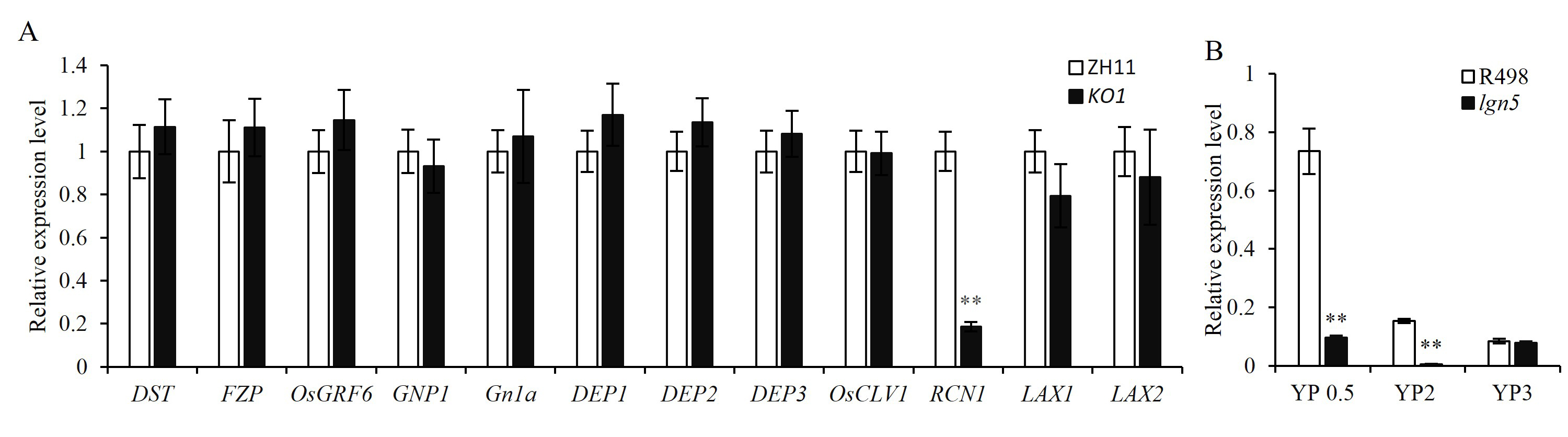


**Supplementary Figure S5. The transcription levels of the genes related to grain number in wild type and mutants.**

**(A****)** The expression levels of the genes related to grain number in the 0.5 cm young panicles of ZH11 and *KO* line. Data are given as means ± SD of three biological replicates. ** indicates P<0.01 by the Student’s *t*-test. **(B)** The expression levels of *RCN1* in the developing young panicles in R498 and *lgn5.* YP0.5, YP2 and YP3, young panicles 0.5, 2 and 3 cm in length. Data are given as means ± SD of three biological replicates. ** indicates P<0.01 by the Student’s *t*-test.


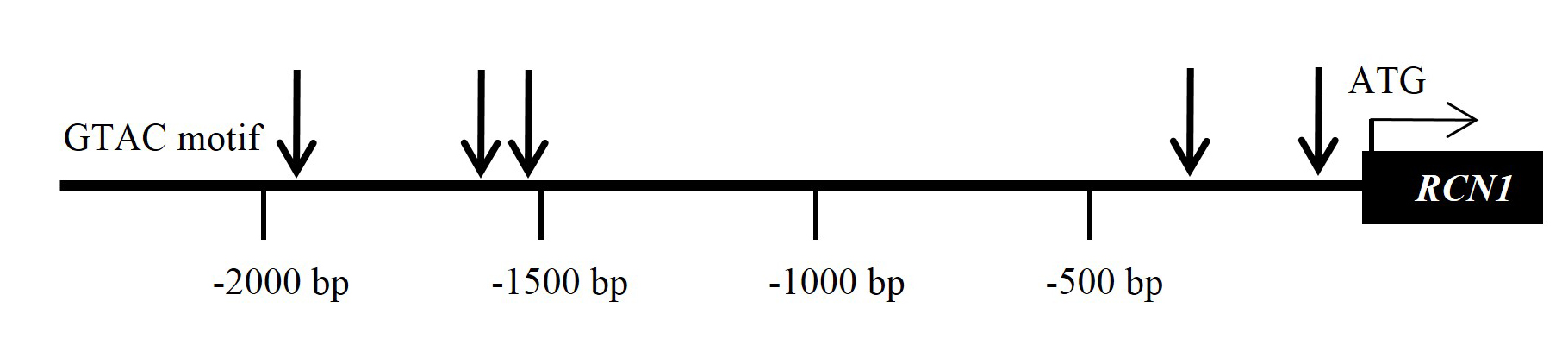


**Supplementary Figure S6. Schematic diagram of *RCN1* gene structure.**

The arrows indicate the position of GTAC motif. The black box shows the genome of *RCN1* gene.
